# Does SMS-Support Make a Difference? Effectiveness of a Two-Week Online-Training to Overcome Procrastination. A Randomized Controlled Trial

**DOI:** 10.3389/fpsyg.2018.01103

**Published:** 2018-07-05

**Authors:** Marcus Eckert, David D. Ebert, Dirk Lehr, Bernhard Sieland, Matthias Berking

**Affiliations:** ^1^Institute of Psychology, Leuphana University of Lüneburg, Lüneburg, Germany; ^2^Institute of Psychology, Friedrich-Alexander-University Erlangen-Nürnberg, Erlangen, Germany

**Keywords:** procrastination, online-training, SMS support, adherence, Rubicon model

## Abstract

The primary purpose of this randomized controlled trial (RCT) was to evaluate the efficacy of an unguided, 2-week internet-based training program to overcome procrastination, called ON.TOP. Because adherence is a typical problem among individuals who tend to procrastinate, especially with internet-based interventions, the secondary purpose of the present study was to investigate whether adding SMS support increases subjects’ frequency of engagement in training. In a three-armed RCT (*N* = 161), the effects of the intervention alone and intervention with daily SMS-support were compared to a waiting list control condition in a sample of students. The primary outcome of interest was procrastination. The secondary outcome of interest was the extent of training behavior. Baseline (T0), immediate post-treatment (T1) and 8-week post-treatment (T2) assessments were conducted. Results indicated that procrastination decreased significantly only with intervention group with daily SMS support, relative to control. Moreover, incorporating SMS support also may enhance extent of training behavior.

## Introduction

Procrastination is a common self-regulatory failure that refers to a person’s inability to initiate or pursue a given goal. It is defined as the voluntary delay of an important activity, even though this activity is intended and/or necessary, and despite the expectation of potential negative consequences (Klingsieck, 2013). The prevalence of procrastination is extremely high. Findings indicate that up to 70% of college students procrastinate (Ellis and Knaus, 1977; Schouwenburg, 1995; Steel, 2007), and almost 50% procrastinate consistently and problematically (Solomon and Rothblum, 1984; Day et al., 2000; Onwuegbuzie, 2000). In addition to being endemic during college, procrastination is also widespread in the general population, chronically affecting 15–20% of the adult population (Harriott and Ferrari, 1996). Although these prevalence numbers refer to self-reported procrastination and not to clinical observations, it can be definitely stated that problematic procrastination is a widespread phenomenon.

Procrastination is a common issue in both work-related and academic contexts (e.g., Steel, 2007). Typical task characteristics that increase the risk of procrastination include how difficult and unattractive a task is, as well as aversive emotional states that can be cued by the task (Blunt and Pychyl, 2000). Typical individual traits that are associated with procrastination are weak impulse control, lack of persistence, lack of work discipline, lack of time-management skills, and the inability to work methodically (Schouwenburg, 2004), as well as deficits in emotion regulation skills (Eckert et al., 2016). For many people, procrastination results in various negative consequences (Pychyl and Flett, 2012) including poor academic and overall performance (e.g., Steel, 2007); negative health behaviors, like postponing required healthcare seeking (e.g., Sirois et al., 2003; Stead et al., 2010); financial disadvantages related to the delayed filing of taxes (Kasper, 2004); and inadequate financial provisions for retirement (Akerlof, 1991; O’Donoghue and Rabin, 1999). Furthermore, studies have found that procrastination decreases a person’s sense of well-being (Lay and Schouwenburg, 1993; Tice and Baumeister, 1997). For example, many adults have regrets that stem from their chronic procrastination across various life domains (Ferrari et al., 2009).

### Factors Affecting Procrastination

There is evidence that procrastination is caused and maintained by a variety of factors, which include the lack of intention building (Owens et al., 2008), poor planning and time management (Lay and Schouwenburg, 1993; Specter and Ferrari, 2000), and time discounting (Howell et al., 2006). Time discounting refers to the tendency individuals have to discount future reward depending on the interval between the activity and reward. A greater interval reduces the motivational value of the reward (Steel and König, 2006). In addition, affective obstacles (Sirois, 2014) like distress (Pychyl et al., 2000; Tice et al., 2001), anxiety (Rothblum et al., 1986), and low positive affect (Ferrari and Díaz-Morales, 2007; Sirois and Pychyl, 2013) mislead individuals into procrastinating to “repair” their mood. Moreover, Wohl et al. (2010) found that forgiving oneself for procrastinating over a specific task decreases subsequent procrastination by decreasing negative affect. Negative affect increases rumination on the transgression of procrastination, and vice versa (Thompson et al., 2005). To achieve short-term mood repair, individuals seek pleasant distractions and postpone tasks they should be doing. But forgiveness “allows the individual to move past their maladaptive behavior” (Wohl et al., 2010, p. 806) and focus on the next task. Their dysfunctional need for procrastination to repair their mood seems to be reduced. Consistent with this, Eckert et al. (2016) showed that one’s ability to cope adaptively with aversive emotions reduces one’s subsequent likelihood of procrastination.

Another equally-important factor that is believed to increase procrastination is a lack of self-reinforcement (e.g., Ferrari and Emmons, 1995). If tasks are boring or fail to induce a positive affect, individuals with procrastination problems tend to seek pleasant distractions. However, individuals who reinforce themselves for doing the boring or aversive task tend to report less procrastination (Ferrari and Emmons, 1995). Consequently, improving these aforementioned factors should subsequently decrease procrastination.

It can therefore be summarized that (1) lack of intention building, (2) lack of planning and time management, (3) difficulties initiating and maintaining a certain action caused by emotional obstacles, poor mood or delayed gratification, and (4) the absence of self-reinforcement and negative self-evaluations, including low self-efficacy expectations, all are associated with procrastination.

### Interventions to Reduce Procrastination

Due to the fact that procrastination causes a lot of problems (e.g., academic impairment, financial problems), interventions to overcome or reduce procrastination are needed. Regarding behavioral interventions, stimulus control provides strategies removing aspects that might interfere with the task (Mulry et al., 1994). In his meta-analysis, Steel (2007) found that interventions that foster automaticity reduced procrastination. Because procrastination is related to avoiding behavior, gradually exposing individuals to aversive activities seems to reduce procrastination (Brown, 1991). In order to overcome lack of commitment with the task, adequate goal setting increases motivation (Boice, 1989).

Since irrational believes and attitudes, like perfectionism, fear of failure, and self-doubt, promote procrastination cognitive interventions, like restructuring, raising self-esteem, and behavioral experiments that facilitate corrective experiences, may reduce procrastination (Rozental and Carlbring, 2014). Glick and Orsillo (2015) found effects of acceptance-based behavioral therapy on procrastination.

Few clinical trials have examined the efficacy of treatment interventions for procrastination (Rozental and Carlbring, 2014). For example, Höcker et al. (2013) reduced procrastination using intervention modules that focused on starting tasks on time, formulating realistic plans of action, and restricting working times. In student samples interventions containing planning and time-management (Schmitz and Wiese, 2006; Häfner et al., 2014) as well as self-instruction methods, such as to stop negative thoughts or positive self-talk (Schmitz and Wiese, 2006), reduced procrastination significantly. In contrast to face-to-face interventions, internet-based interventions are cost-effective in the treatment of a wide range of problems (Rozental et al., 2015). Surprisingly, only a few randomized controlled trial (RCT) have investigated internet-based interventions in this field (Wäschle et al., 2014; Rozental et al., 2015), clearly justifying the need for further RCTs of Internet-based interventions to decrease procrastination.

Rozental et al. (2014) found that a 10-week internet-based intervention providing psychoeducation and several techniques in 10 modules reduced procrastination with a medium effect size. The techniques included behavioral activation, graded exposure, behavioral experiments, identifying and testing rigid beliefs and assumptions, and stimulus control. In a three-armed randomized controlled study, 150 participants were randomized either on treatments with therapist contact (guided), treatment without therapist contact (self-guided), or wait-list control. Compared with the wait-list control, the guided condition revealed greater effect sizes than the self-guided condition.

### First Purpose of the Present Study

Internet-based interventions provide many advantages like improved access, cost-effectiveness, local and time independence. Thus, the first purpose of the present study is to develop and to evaluate a brief self-guided internet-based intervention to reduce procrastination utilizing the advantages. In the following section we describe factors affecting procrastination. Based on these factors, we provide the Rubicon-model as heuristic for the development of an intervention. But internet-based interventions have also disadvantages like decreased adherence compared to face-to-face treatments (Richards and Richardson, 2012; Andersson and Titov, 2014). That followed, we describe factors affection adherence and we suggest text messages (SMS) as intervention to reduce the problems with adherence in internet-based interventions. Thus, the second purpose of the present study is to investigate whether additional SMS support increased adherence to (as well as efficacy of) the internet-based intervention. In contrast to the intervention of Rozental et al. (2014), the present study investigated the effectiveness of a 2-week intervention which provides SMS support instead of therapist contact. With regard to the content, the Online-Training to Overcome Procrastination (ON.TOP) is orientated on the Rubicon-model from Heckhausen and Gollwitzer (1987) as shown below.

### The Rubicon-Model as a Heuristic for Developing an Intervention

Addressing factors that cause and maintain procrastination, the Rubicon-model (Heckhausen and Gollwitzer, 1987) was used as a heuristic for developing interventions to reduce procrastination. The Rubicon-model is a theory of action regulation (Heckhausen and Gollwitzer, 1987) that differentiates four motivational and volitional phases: (1) intention-building; (2) time-management planning and realistic goal setting; (3) shielding the intended action from distractions; and (4) evaluating the process and the results of completed activities. The first phase is pre-decisional. It emphases the process of pondering the “pros and cons of one’s wishes […] by assessing the desirability of expected outcomes and the question of feasibility” (Achtziger and Gollwitzer, 2007, p. 769). This leads into constructing intentions to act. For the purpose of acting successful, the pre-actional second phase focuses on planning when, where and how one must work toward the goal. It includes several volitional processes. In the actional third phase, goal-directed behaviors must be initiated and maintained by volitional processes like emotion regulation. Upon completion of goal-directed behaviors, during the post-actional fourth phase, the outcome must be evaluated. This evaluation influences future intention building by influencing self-efficacy and action-outcome expectations. This heuristic model addresses factors that both cause and maintain procrastination; as such, it provided the basis for developing our intervention to reduce procrastination.

#### Pre-decisional Phase

In line with several theorists (e.g., Ajzen and Fishbein, 1969; Ajzen, 1991; Bandura, 2005; Sniehotta et al., 2005; Schwarzer, 2008), the Rubicon-model emphasizes the role of intention-building for successful action-regulation and self-regulation. Postponing intention-building is called decisional procrastination that impairs also performance (Ferrari et al., 1995). Individuals who scored high on decisional procrastination have been found to “search more information about the chosen alternative […]” (Ferrari and Dovidio, 2000). As a result, they often shirked intention-building. Hence, promoting adaptive decision strategies and generating behavioral intentions may reduce decisional procrastination (Owens et al., 2008; Shin and Kelly, 2015).

#### Pre-actional Phase

Although intention building is necessary for successful action- and self-regulation, on its own it is insufficient. A body of research exists that deals with the intention-behavior gap (Sheeran, 2002). It has been shown that realistic goal-setting — achieved by creating concrete intermediate goals — increases one’s probability of executing intended tasks (van Eerde, 2000). Planning execution conditions increases the probability of goal-directed behaviors. Implementation intentions are simple “if-then” plans that determine when and how a given task should be executed. They promote goal-directed behavior and, by doing so, reduce the gap between intentions and behaviors (Gollwitzer, 1999). In this way, the realistic planning and specification of execution conditions reduce tendencies to procrastinate (Owens et al., 2008).

#### Actional Phase

Despite realistic planning and implementation intentions, emotional obstacles can disturb the execution of intentions (Pessoa, 2009). As described earlier, individuals often procrastinate to repair their mood. Eckert et al. (2016) have shown that emotion-regulation skills can reduce procrastination, and have suggested that emotion-regulating skills may reduce the need for dysfunctional mood repair. Thus, to reduce procrastination, emotion regulation skills could shield intended actions from distractions, like emotional obstacles.

#### Post-actional Phase

Self-reinforcement during aversive or effortful behaviors increases the likelihood that the desired behavior will occur (Gokee-LaRose et al., 2009), whereas the absence of self-reinforcement increases the likelihood of procrastination (Ferrari and Emmons, 1995). Moreover, recognizing one’s own ability to initiate and maintain intended behaviors, and to recognize the positive effects of this behavior, increases self-efficacy expectations (Bandura, 1977). Several studies have revealed that establishing self-efficacy expectations prevents procrastination (Steel, 2007; Klassen et al., 2008). Thus, positive self-evaluations relating to one’s intention-behavior relationship and related positive effects may help individuals to decrease their tendencies to procrastinate.

Past findings indicate that individuals who score high on procrastination postpone activities when there are obstacles to overcome (Steel, 2007). For example, such individuals are more likely to postpone writing a letter if they have to clean up their desk beforehand. Taking this into consideration while developing an intervention to reduce procrastination, one should incorporate fewer motivational and volitional obstacles to initiate the intervention. With respect to time-discounting effects, offering a short intervention with immediately-noticeable effects could be beneficial. Moreover, the high accessibility of an internet-based intervention might also reduce motivational and volitional obstacles.

Unfortunately, internet-based interventions often involve problems of adherence (Richards and Richardson, 2012). When face-to-face and internet-based interventions are compared, most of the latter are associated with less treatment adherence (Christensen et al., 2009). Typically, individuals scoring high for procrastination are less adherent to any form of treatment (Ferrari et al., 1995). Thus, despite all the advantages of internet-based interventions — including their easy access, independence of time and place, and personal anonymity — individuals who tend to procrastinate may be especially unlikely to adhere. However, the outcomes of such interventions also depend on the extent to which participants engage in active training. Thus, those striving to develop any internet-based intervention targeting procrastinators should incorporate components that increase their adherence.

### Factors Affecting Adherence to Online-Based Interventions

Adherence to treatment is defined as the extent to which the participant of an intervention coincides with the prescribed treatment (Urquhart, 1996). Research has identified certain factors that increase adherence to internet-based interventions. These include tailored feedback by e-coaches (Cugelman et al., 2011); program interactivity (Hurling et al., 2006); an enriched training environment that includes features like multimedia presentations and audio-exercises (Webb et al., 2010); and text messages as reminders (Fjeldsoe et al., 2009; Krishna et al., 2009). In particular, short-message service (SMS) support appears to enhance the effectiveness of internet-based health interventions (Webb et al., 2010), having been applied in the research literature to fulfill a variety of functions. For example, Kamal et al. (2015) increased medical adherence among stroke patients by providing reminders and health information by SMS. Similarly, Koshy et al. (2008) reminded ophthalmology outpatients of appointments and, thereby, increased their adherence. Kaptein et al. (2012) implemented successfully social influence strategies as prompts, via SMS, in order to reduce snacking. However, the success of this strategy seems to depend upon individually-tailored messages, with the effectiveness of non-tailored messages less apparent.

Another reason SMS increases adherence may be seen with the so-called “foot-in-the-door” (FITD) technique. This technique works by first inducing a “yes” response for a small request. This primary “yes” increases the probability of receiving an affirmative response to subsequent, greater requests. Several investigators have found evidence that the FITD technique influences behavior (e.g., Freedman and Fraser, 1966; Guéguen and Fischer-Lokou, 1999; Guéguen et al., 2008), and it appears to be an effective strategy for real-world interventions (Guéguen, 2002; Cugelman et al., 2009; Grassini et al., 2013). As such, to increase adherence, very small exercises provided daily via SMS may be appropriate. These mini-exercises may be the proverbial “foot in the door” and increase the probability of later engagement with the intervention. However, to the best of our knowledge, no prior research has systematically investigated whether the FITD technique has any role increasing adherence through SMS support.

### Goals of the Present Study

The present study was designed to evaluate the internet-based intervention ON.TOP, which was developed in accordance with the Rubicon model (Heckhausen and Gollwitzer, 1987). Relative to the first Internet-based intervention created to reduce procrastination, reported by Rozental et al. (2015), ON.TOP focuses more on strategies to cope adaptively with emotional obstacles cueing procrastination; for example, strategies to tolerate and modify aversive emotions (Eckert et al., 2016). In this way, the current study will enrich the research literature on Internet-based interventions targeting procrastination.

Recalling that Internet-based interventions often must confront problematic adherence, a second aim of this study was to investigate if daily SMS support increases adherence to and/or the efficacy of the intervention. Thus, every day, participants in one of the two intervention arms of the study received two short exercises via text messages (SMS support). These exercises were intended to motivate subjects in various ways, including use of the foot-in-the-door technique by which affirmative responses to simple requests were used as a springboard to further engagement in anti-procrastination training exercises.

### Hypotheses

We hypothesized that (1) participation in the intervention groups would lead to greater reductions in procrastination at the time of post-program measurement and at 8-weeks post-treatment follow-up, relative to that observed in waiting list controls (WLC); (2) participants receiving SMS support would report a higher frequency of engagement in anti-procrastination training than participants with no SMS support; and (3) combining the ON.TOP program and daily SMS support would generate a greater reduction in procrastination than ON.TOP without SMS support.

## Materials and Methods

### Design and Timeframe

This study was a three-armed RCT that compared (1) an internet-based unguided intervention administered alone (I_A_), (2) the same internet-based intervention, but with additional guidance via SMS texts (I_SMS_); and (3) waiting list controls (WLC). Variables were measured immediately prior to treatment (T0), immediately after the 2-week program (T1), and 8 weeks post treatment at a final follow-up assessment (T2). Based on the findings of Rozental et al. (2015), and because our intervention was newly-developed, we expected that the intervention would have an effect of medium size (Cohen’s *d* = 0.50). Accordingly, a sample size of *N* = 161 was required to detect a difference between the three treatment groups. This estimate was based on intention-to-treat analyses with α = 0.05 and 1 – β = 0.95 in a two-tailed test.

To keep all subjects’ workload equal and constant, thereby eliminating as a source of bias differences in level of activity, the intervention was administered during a lecture period. Interested individuals were recruited into the study and randomized to one of the three study groups from October to December 2014. The last post-treatment measurement took place in December 2014. The last final-follow-up measurement was completed in February 2015. In February 2015, students were entering a part of their school curriculum when writing examinations and papers replaced lectures.

All procedures were approved by the Institutional Review Board at Leuphana University of Lüneburg, Germany.

### Procedures and the Sample

Since half of students report serious procrastination problems (Day et al., 2000), we decided to recruit subjects from a student population. To recruit such students, we distributed information (a) via the internal communication system at Leuphana University of Lüneburg, (b) via several helplines for students at three German universities (in Hannover, Hildesheim, and Lueneburg), and (c) via the Moodle Communication System used by the co university in Hagen (Germany). All participants reported to be university students. All individuals completing the baseline online survey (T0) and providing informed consent were included. After completing the baseline survey, participants were randomized in Excel, using the RandBetween function, which automatically assigned the number 0, 1, or 2 to each ID number, indicating allocation to the I_A_, I_SMS,_ or WLC group, respectively.

**FIGURE 1:**
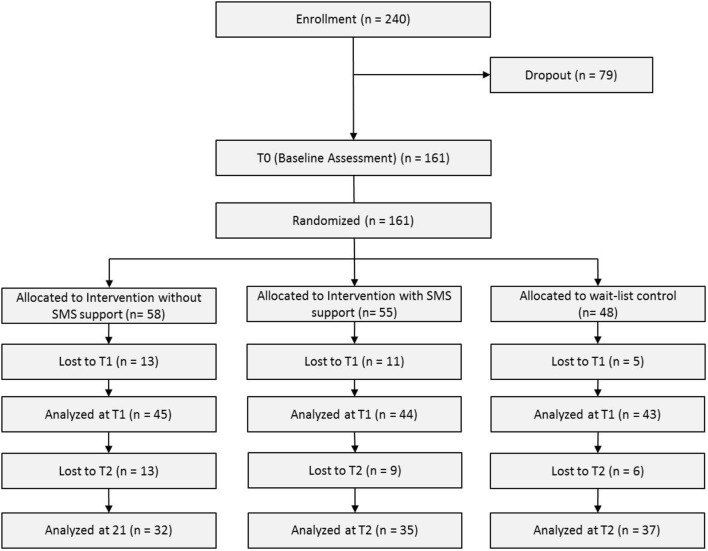
CONSORT flow diagram.

Participants who were randomized to receive either I_A_ or I_SMS_ received access to the internet-training program via e-mail. Additionally, subjects in the I_SMS_ group also received two text messages daily (SMS support). Waiting list controls merely received information about the progress of the study. Two weeks after completing the baseline questionnaire, and immediately after completion of the 2-week intervention, all participants were asked to complete a second questionnaire (T1), followed by a final follow-up questionnaire 8 weeks after completing the second questionnaire. At that point, subjects in the WLC group were granted access to the ON.TOP program. At all three data collection points (T0, T1, T2) participants completed the General Procrastination Scale (Klingsieck and Fries, 2012). At baseline, socio-demographic data also were collected; at the immediate post-treatment assessment (T1), subjects were asked to indicate the frequency of their engagement with the training program over the preceding 2 weeks. **Figure 1** shows the flow diagram of this study.

A total of 161 students were randomized to either the I_A_ (*N* = 58; 67.2% females), the I_SMS_ (*N* = 55; 74.5% females), or WLC group (*N* = 48; 81.3% females). Overall, 119 of the subjects (73.9%) were women, and the average age was 28.4 years (*SD* = 8.9), ranging from 19 to 62 years; this broad age range is explained by our inclusion of several mature students who had returned to school later in life. Regarding the sample’s career choice, 63 participants (39.1%) studied educational science or related subjects, 42 (26.1%) studied psychology, 12 (7.5%) studied economic or related science, 7 (4.3%) studied engineering, 5 (3.1%) studied cultural studies, 3 (1.9%) studied politics, 3 (1.9%) studied informatics, 3 (1.9%) studied environmental sciences, 3 (1.9%) studied digital media and another studied jurisprudence (0.6%). Nineteen participants (11.8%) did not report their subject. Twenty-seven (16.8%), five (3.1%), and three (1.2%) of the 161 subjects stated that they had been diagnosed with depression, anxiety, or attention deficit-hyperactivity disorder (ADHD), respectively. No differences in the three treatment groups were apparent for gender distribution (χ^2^ = 2.69; *p* = 0.26) or age (*F* = 1.049; *p* = 0.35). Similarly, there were no inter-group differences in the percentage with depression (χ^2^ = 0.979; *p* = 0.61), anxiety (χ^2^ = 3.488; *p* = 0.18), or ADHD (χ^2^ = 0.019; *p* = 0.99).

### Intervention

**FIGURE 2:**
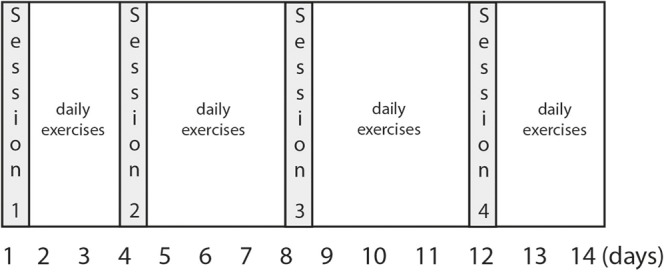
Timetable of the ON.TOP sessions.

ON.TOP combines already-available, well-established therapeutic techniques to reduce procrastination. It consists of four sessions which includes videos, audio exercises (relaxation or imagination exercises), and written material. To reduce potential obstacles to starting or maintaining the intervention, (1) all text-based information was also provided in audio format, and (2) no session exceeded 30 min in duration. To minimize time-discounting effects, participants were asked to reward themselves with some form of positive reinforcement every evening for every successful attempt they had made that day to decrease procrastination, no matter how small the attempt. **Figure 2** display the timetable of the sessions.

The Rubicon Model (Heckhausen and Gollwitzer, 1987) was applied as the theoretical framework. It includes the following phases: (a) motivational phase (intention building), (b) pre-actional phase (planning), (c) actional phase (realizing), and (d) post-actional phase (evaluating). The 2-week intervention was therefore comprised of four sessions.

During the first session (intention building), participants learned about the relevance of decision-making (Steel, 2007) by watching a psychoeducation video. Keller et al. (2011) found that enhancing active choice fosters desired behaviors. Thus, in the first session, participants were trained to actively decide whether to implement an intention or not. To achieve this, they were asked to identify one of their daily tasks that they were most likely to delay completing. To generate the intention to complete this task, they then were asked to contrast the long-term benefits of executing the task against the long-term costs of avoiding or postponing the task (e.g., failing an examination). They also were asked to compare the short-term costs of executing the task (e.g., boredom) and short-term benefits of avoiding or postponing it. Finally, once they had determined if there were any good reasons why the task should be postponed or avoided, participants were invited to actively decide to execute the task and accept the short-term costs and protocol them in a diary. The diary was provided in PDF format. Decision- and goal setting strategies are often considered suitable for addressing problems of procrastination from cognitive behavior therapy (CBT) (Steel, 2007).

In the second session (planning), participants learned about two principles of planning. The first of these is realistic goal setting, achieved by establishing concrete intermediate goals, a process that tends to increase one’s probability of executing the ultimately-intended task (van Eerde, 2000). One example of this is suggesting that students plan to read and summarize a single chapter per day, instead of studying until they can no longer continue. The second principle involves implementing simple “if-then” plans to determine when and how to execute the task and, thereby, reduce the gap between intentions and behaviors (Gollwitzer, 1999). Previous research indicates that applying implementation intentions may reduce procrastination (van Hooft et al., 2005; Gollwitzer and Sheeran, 2006). Like the first session, subjects were asked to identify one of their daily tasks which they were most likely to procrastinate against doing. If they decided to actively execute the task, they were encouraged to plan task execution by setting up realistic (sub-) goals and applying if-then plans and protocoling them in a PDF diary.

In the third session (realization), subjects learned how to overcome affective obstacles that created gaps between their intentions and behaviors (Eckert et al., 2015). Eckert et al. (2016) have shown that two emotion-regulation strategies are effective at reducing procrastination: tolerating aversive emotions and modifying them. The third session of the ON.TOP program focused on these two strategies. As in the previous sessions, participants were asked to choose one daily task they were most likely to try to delay completing, to actively decide whether to execute the task, and then to plan task execution. They then were asked to (a) identify and label which aversive emotions were cued by the task, then (b) tolerate, and finally (c) modify the aversive emotion. As per Berking and Whitley (2014), the strategy to tolerate aversive emotions included intentionally permitting aversive emotions to be present, reminding oneself of one’s toughness and resilience, and finally reminding oneself of (or increasing) one’s affective commitment with the task. The modification strategy of aversive emotions consisted of first practicing a short relaxation exercise, then reappraising the harm and probability of the potential threat, and lastly deciding whether to execute the task. After completing the chosen task, participants evaluated how successfully they had coped with their aversive emotions to increase their emotional self-efficacy. The emotion regulation strategies were presented via audio files.

During the fourth and final session (evaluating), expectations of self-efficacy were fostered, as self-efficacy expectations are a relevant negative predictor of procrastination (Steel, 2007). Hence, participants evaluated situations in which they were able to reduce procrastination successfully (Sirois, 2004). This may increase both self-efficacy expectations and self-reinforcement to subsequently reduce procrastination. Additionally, they were asked to reflect on why previous strategies had been unsuccessful, so they might work to improve or replace them.

### SMS Support

Subjects in the I_SMS_ group received two brief messages each day, all delivered via SMS on their own cellphone. Each message gave them a short, simple exercise or task to complete, none requiring more than 30 s. The objective of these exercises always was aligned with the content of the current intervention session. Examples of SMS content are: “Which task are you most likely going to postpone today? What are the consequences if you decide not to do the task? Are there any good reasons against this decision?” (Session 1). “Which task are you most likely going to postpone today? If you have decided to do this task, set the time when you will begin now” (Session 2). “Remember a situation in which you overcame procrastination successfully. Try to remember this feeling of success before you will start another aversive task” (Session 3). “In this training program, you have learned how to overcome procrastination. Now it is time to look back and acknowledge your own success” (Session 4). Therefore, participants in both intervention groups followed a timetable that indicated to them when to start and when to end each session (Session 1: day 1–3; Session 2: day 4–7; Session 3: day 8–12, and Session 4: day 13 and 14). Participants in the I_SMS_ arm received reminders to start their next session via SMS.

In each of the examples listed above, note how every SMS message contains a small, simple task or exercise (e.g., set a time), which motivates participants to complete further training. The threshold for complying is very low. Using this foot-in-the-door (FITD) technique, each “yes” to a small request was hypothesized to increase the probability that the subject would be adherent and engage in later, more time-consuming exercises.

### Measurements

#### Procrastination

Procrastination was measured with the German short version of the General Procrastination Scale (GPS; Lay, 1986; German version: Klingsieck and Fries, 2012). The GPS is a self-report instrument with nine items that utilize a 4-point Likert-type response scale (1 = *extremely uncharacteristic* to 4 = *extremely characteristic*). Four of the nine items are inversed. A sample item is “I often find myself performing tasks that I had intended to do days before” (Lay, 1986). Due to the lack of psychometric validity, Klingsieck and Fries (2012) revised the original scale factor-analytically. An average score was obtained by summing the individual scores for all nine items, and then dividing by nine. In the present study, the internal consistency of the GPS was good (α = 0.83).

#### Frequency of Engagement

At the time of their immediate post-treatment assessment, all participants were asked to rate the frequency with which they used the training program on a 6-point Likert-type scale, by answering the question, “How often did you engage in ON.TOP practice exercises? (1 = not at all; 2 = rarely; 3 = sometimes; 4 = often; 5 = very often; 6 = daily)”.

### Statistical Analysis

In the current paper, we present the results of intention-to-treat (ITT) analyses performed using the statistical software program SPSS, version 22 (IBM Corp, Armonk, NY, United States). Due to dropout rates of 18.6% (post-treatment) and 34.8% (final follow-up), we also decided to report the results of completer analyses, only analyzing data from those subjects who completed the program. Reported test-values are two-sided, with the threshold for statistical significance set at 0.05.

#### Missing Data

All participants completed the baseline assessment. A Markov Chain Monte Carlo multivariate imputation algorithm (SPSS 22) with ten estimations per missing value was used to replace all missing post-treatment and follow-up data (Schafer and Graham, 2002). With multiple imputations (MI), predictors are defined, which leads to missing value estimates by regression analyses. With MI, predictors are defined in a way that leads to reasonable estimates of missing values. For each missing value estimate, this included using each subject’s other pre-, post-, and 8-week follow-up values, as well as age and gender norms within the sample. In order to use the imputations for further analyses in SPSS, we conducted for all missing values an aggregation of all 10 estimations.

#### Treatment Efficacy

To assess treatment efficacy, outcome values for the I_A_ and I_SMS_ groups were compared against WLC values. As a first step, analysis of variance (ANOVA) with repeated measures (pre, post, follow-up) included both intervention groups and the WLC was calculated. To contrast the effectiveness of treatment relative to no treatment (WLC), Dunnett-T *post hoc* tests (<WLC) was conducted. We also calculated effect sizes (Cohen’s *d*) between groups at T2 and with groups. In a second step, we finally compared both intervention groups against each other by conducting a 2 × 3 ANOVAs.

Additionally, we conducted a 3 × 3 ANOVA (with repeated measures), only including those who had completed the program and full follow-up (completer analysis).

#### Frequency of Engagement

Given that the frequency of engagement was measured using a single-item self-rating scale, we analyzed differences between the two intervention groups with Kruskal–Wallis (non-parametric) tests. On initial analysis, all participants were included, except the two who failed to report their frequency of engagement (*N_A_* = 32; *N_SMS_* = 33). Considering that the SMS messages referenced the online content, we assumed that the combination, but not SMS alone, would influence the frequency of engagement. For example, if the SMS suggest “Get your anchored positive emotion before you start the task,” it is necessary that participants anchored positive emotions in the online-session previously. Thus, during a second *post hoc* analysis, we were forced to exclude eight participants in each group, as they rarely or never engaged in ON.TOP (*N_A_* = 24; *N_SMS_* = 25). All analyses assessing the frequency of engagement were completer analyses.

## Results

### Missing Data

At the end-of-treatment assessment (T1), missing data had to be accommodated for 17.4% of the 161 subjects overall, including *N* = 13 (22.4%) among I_A_, *N* = 11 (20.0%) among I_SMS_, and *N* = 5 (10.4%) among WLC subjects. At 8-weeks follow-up (T2), corresponding percentages were 35.4, 44.8, 36.4, and 22.9%. At T1, the percentage with missing data did not differ significantly between the groups (χ^2^ = 2.668; *p* = 0.26), while the three treatment groups were marginally different at T2 (χ^2^ = 5.547; *p* = 0.06). Comparing the two intervention groups separately against the WLC group, a significant difference was apparent between I_A_ and WLC (χ^2^ = 5.549; *p* = 0.02), but not between I_SMS_ and WLC (χ^2^ = 2.203; *p* = 0.20).

Comparing those how complete and those how dropped out, we found no difference concerning age (*M*_dropout_ = 27.7; *SD*_dropout_ = 8.69; *M*_completer_ = 28.8; *SD*_completer_ = 8.96; *t*_1,158_ = 0.090; *p* = 0.93), concerning gender (71.9% of those who dropped out were female 75.0% of those who completed were female; *N* = 161; χ^2^ = 0.180; *p* = 0.71), and concerning procrastination (*M*_dropout_ = 3.3; *SD*_dropout_ = 0.49; *M*_completer_ = 3.2; *SD*_completer_ = 0.49; *t*_1,158_ = 1.170; *p* = 0.24).

### Treatment Efficacy

These results based on intention-to-treat analyses (ITT-analyses). In order to compare the three treatment arms simultaneously, the means of the GPS of each point of measurement as well as the group factor (WLC; I_A_ versus I_SMS_) were included in an ANOVA with repeated measures. The main effect of measurement time (*F*_4,316_ = 54.71, *p* < 0.001, $\eta_{p}^{2}$ = 0.415) as well as the interaction term ‘time × condition’ was significant (*F*_4,316_ = 7.91; *p* < 0.001, $\eta_{p}^{2}$ = 0.110) were significant. Means for the GPS decreased over time in all three groups. Means and standard deviations for both ITT-Analysis and Completer-analyses are displayed in **Table 1**. *Post hoc* contrast at T1 revealed no significant differences between the groups. At T2, Dunnett-T indicated significant differences between WLC and I_SMS_, but not between WLC and I_A_ (see **Table 2**).

**Table 1:** Means and standard deviations of the General Procrastination Scale (GPS).

		WLC (*N* = 48/37)		I_A_ (*N* = 58/32)		I_SMS_ (*N* = 55/35)		Statistics		
		*M*	*SD*	*M*	*SD*	*M*	*SD*	*F*	*P*	η^2^
**Intention-to-treat analyses**		
	T0	3.11	0.56	3.23	0.45	3.28	0.46	7.910	0.00	0.110
	T1	3.02	0.57	2.97	0.49	2.85	0.61			
	T2	3.00	0.61	2.82	0.51	2.64	0.64				
**Completer-analyses**		
	T0	2.81	0.75	3.07	0.43	3.04	0.59	10.162	0.00	0.173
	T1	2.78	0.79	2.76	0.58	2.49	0.82			
	T2	2.79	0.76	2.62	0.64	2.11	0.80			

**Table 2:** *Post hoc* test Dunnett-T (<WLC) in order to contrast effects of intervention groups at follow-up assessment.

			T1		T2	
	Group I	Group J	Difference I – J	*P*	Difference I – J	*P*
Dunnett-T (<WLC)	WLC	I_A_	0.06	0.44	0.16	0.14
	WLC	I_SMS_	0.17	0.11	0.35	0.00

**FIGURE 3:**
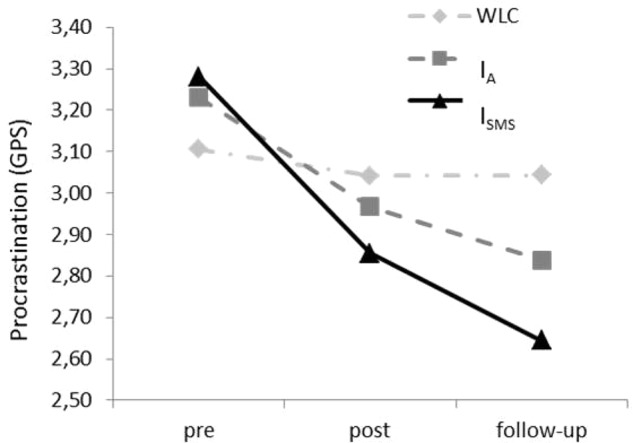
Changes in the extent of procrastination (GPS) from pre-measurement (T0) to post-measurement (T1) to 8-week follow-up (T2) in the intervention groups (I_A_ and I_SMS_) and the waiting list control (WLC) group.

Effect sizes for I_SMS_ seemed to be stronger than effect sizes for I_A_. Comparing WLC against the two intervention groups at T2, we calculated a small effect size for I_A_, for GPS (*d* = 0.29; 95% CI [-0.703, 0.063]), but a medium effect size for I_SMS_, again for the GPS (*d* = 0.57; 95% CI [-0.964, -0.182]). Calculating effect sizes within groups, procrastination seemed to be more reduced by I_SMS_ (*d* = 1.15; 95% CI [-1.719, -0.578]) than by I_A_ (*d* = 0.85; 95% CI [-1.390, -0.315]). **Figure 3** reveals changes in procrastination as measured on the GPS between T0 and T2.

In order to investigate if additional SMS-support makes a difference, we comparing both intervention groups directly against each other (I_A_ versus I_SMS_). A 2 × 3 ANOVA (with repeated measures) revealed a significant interaction term between group and time (*F*_2,111_ = 3.67, *p* < 0.05, $\eta_{p}^{2}$ = 0.062).

Regarding effectiveness, when only completers were included, group differences were still significant (see **Table 1**). Effect sizes at the time of final follow-up (T2) were similar to those we identified on intention-to-treat-analyses: I_SMS_: *d* = 0.87; 95% CI [-1.361, -0.393]; I_A_: *d* = 0.24; [-0.706, 0.222]; *d* = 0.28; [-0.745, 0.184]. Moreover, comparing the efficacy of both intervention groups directly via additional ANOVA with repeated measures, the interaction term ‘time × condition’ was significant for GPS (*F*_2,63_ = 5.31; *p* < 0.01, $\eta_{p}^{2}$ = 0.144).

### Frequency of Engagement

Comparing I_A_ (*M* = 2.6; *SD* = 1.17; *N* = 32) and I_SMS_ (*M* = 3.0; *SD* = 1.60; *N* = 33), we identified no significant difference in the frequency of engagement with the treatment program (*Kruskal–Wallis* = 0.57, *p* > 0.10). However, after we excluded participants who engaged in treatment rarely or not at, those in the I_SMS_ group (*M* = 4.1; *SD* = 1.25; *N* = 25) scored significantly higher than those in the I_A_ group (*M* = 3.4; *SD* = 0.87; *N* = 24) (*Kruskal–Wallis* = 4.08, *p* < 0.05).

## Discussion

The present study evaluated the efficacy of an internet-based training program to reduce procrastination. Two intervention groups were compared against a waiting list control group. When we included both intervention groups and WLC in a three-armed ANOVA, Dunnett-T test indicated that only I_SMS_ seemed to reduce procrastination significantly compared to the WLC. We found that the intervention that included motivational prompts delivered by SMS was clearly more effective at reducing procrastination than being on a waiting list, with intermediate effect size.

Like other unguided internet-based interventions (i.e., interventions to reduce stress or depression), ON.TOP exhibited small to medium effect sizes (Richards and Richardson, 2012). In line with the theoretical assumption and empirical findings of Webb et al. (2010) on the enrichment of internet-based interventions, we found a significantly greater reduction in procrastination among patients who received the intervention with versus without SMS support. When effect-size confidence intervals were evaluated, only the intervention including SMS had 95% confidence intervals that excluded zero. Rozental et al. (2015), assessing a 10-week internet-based program based on cognitive behavior therapy (CBT-I) to reduce procrastination, identified effect sizes between *d* = 0.50 and *d* = 0.81 immediately after treatment. Although it is difficult to compare their and our outcomes, given the two very different populations that were analyzed, our findings seem to be consistent with other previous studies (e.g., Rozental et al., 2015). Moreover, it seems that that the brevity of the intervention ON.TOP was compensated by adding daily SMS support.

How the addition of SMS support might augment the effects of internet-based interventions targeting procrastination remains unclear. For example, whereas Fjeldsoe et al. (2009) investigated the reminder function of text messages, we assume that the effects of SMS support were not completely mediated by reminding subjects to participate in training, but also by the foot-in-door-technique which usually refers to social interaction (Guéguen et al., 2008). For clarity, more research is needed. In line with our assumption, Cugelman et al. (2011) have claimed that interactive elements in online interventions may evoke feelings of real social interaction. Some of our participants mentioned that they almost wanted to respond to the daily SMS messages they received, despite being fully aware that they were generated automatically. Furthermore, they reported that if they had not engaged in the training, they would have felt guilty upon receiving the SMS. This suggests that SMS was perceived as more than just an automatic reminder, but rather as some form of social contact.

Although descriptive statistics indicate that the frequency of engagement was higher in those receiving versus not receiving SMS support, these differences were not significant when analyzing the full sample. These findings are contrary to those of other investigators, who found that reminders via text messages increased adherence to their program (Fjeldsoe et al., 2009; Webb et al., 2010). This is understandable, when one considers the assumed impact of the SMS support for the intervention, the content of the SMS referencing the content of the online-based training. If participants failed to partake of the training program at all, they were unable to follow the instructions delivered via SMS. Considering the foot-in-the-door hypothesis, it may be more difficult for participants who did not engage at all in the online-training to comply with the small tasks requested via the SMS texts than for those who did engage. Those who engaged at least a little were presumably able to connect the question or task delivered by SMS with content previously learned through the online-training. For those that did not engage in training at all, the SMS messages might have been uninterpretable.

Thus, the foot-in-the-door technique might not work, except in those who already are complying with treatment, at least to some degree. To examine this possibility, we also performed *post hoc* analysis excluding subjects who only engaged in the internet intervention minimally, if at all, and found that adherence with the program was higher among those receiving SMS support. Although we did not systematically investigate whether SMS increased adherence via the foot-in-the-door technique, the findings of the present study provide preliminary support for this assumption. Thus, future research is needed to systematically analyze pre-conditions of the efficacy of SMS support in a sample of individuals with high procrastination scores, and quantify the minimum level of engagement that is necessary to successfully utilize SMS text messaging. To further test the above-noted foot-in-the-door hypothesis, participants should be further randomized to one group that only receives reminders to participate in the online training, or to a second group that receives SMS messages that contain easy tasks or short exercises for them to do, thereby testing the FITB assumption that earning a “small yes” increases the likelihood of further, more extensive compliance and/or participation.

The present study has several limitations. First, procrastination was only assessed with a self-report inventory, without behavioral observations. Although a large body of literature indicates that procrastination often leads to poorer performance (e.g., Steel et al., 2001), some authors suggest that at least some individuals use procrastination as a performance-enhancing strategy (Strunk et al., 2013). Thus, future research should assess procrastination, both by observing behavior and by assessing the harm of procrastination-related consequences. Second, although the questionnaires used in this study were behavior-orientated, they assessed procrastination as a trait-like construct. The aim of the intervention was to change coping strategies with general (trait-like) tendencies that affect behavior and lead to procrastination. Thus, future research should measure this change in coping strategies more appropriately (e.g., by observing specific assignments or academic tasks in combination with structured interviews on how participants deal with postponing and avoidance tendencies). Moreover, in the present study, we assumed only small variations in workload, but this was not actually measured. Future investigators should record workload.

Third, based on post-hoc analysis, we found that SMS support only influenced those who actually engaged in the training exercises. *Post hoc* analyses may be weak evidence. Thus, for a better understanding of the effects of SMS support, a study involving subjects with at least a minimum level of treatment engagement is needed. Moreover, future studies should include SMS messages requesting the completion of minor tasks that are easily understood, even by those who have not participated in the intervention of interest. This could increase the probability that subjects will fulfill the tasks sent to them via SMS messages, even if they have not yet started internet-based intervention. Fourth, in terms of both external validity and generalizability, our findings are constrained to students. Fifth, a WLC decreased the internal validity. Thus, a replication of this study should conduct a placebo control instead. Sixth, eight weeks of follow-up might be considered too brief; more prolonged follow-up to document reduced procrastination over a more extended period of time would have been much more meaningful, and should be incorporated into future studies. This being said, even short-term benefits could be of value, with individuals able to participate in and benefit from online-training even in the short term when there is a pressing need for them to limit their procrastination (i.e., when preparing for examinations). Seventh, in both intervention groups, a sizeable percentage of participants failed to complete the final follow-up assessment. Although we systematically generated estimates to replace all missing data, this may have distorted our results. On the other hand, when we only included those who had completed the study in analysis, the group differences that we had detected earlier on intention-to-treat analysis persisted, and the effect sizes at final follow-up were similar to those of intention-to-treat effect sizes. Unfortunately, we have no data on the reasons subjects elected to drop out of the study. One question that arises is: why were those in the two intervention groups less likely to complete their forms fully than our waiting list controls? One plausible explanation is that participants on the waiting list had been promised access to the intervention once they completed the follow-up assessment, whereas those in the two intervention groups attained no further benefit from completing the final questionnaires. Taking in account that all our study participants were, by necessity, students with procrastination problems, this final failure to complete forms should not come as a huge surprise.

## Conclusion

Our study shows that a short internet-based intervention can help students to reduce procrastination, and that SMS support might increase the intervention’s effectiveness. Since e-coaching is often provided to increase treatment efficacy and adherence in internet-based interventions (Ebert et al., 2014), SMS might be an effective, lower-cost alternative. However, future research should clarify the mechanism mediating the additional impact of SMS-support on decreasing procrastination. It might be beneficial to consider the foot-in-door-technique.

## Ethics Statements

Ethical Review Board of the Leuphana University of Lüneburg, Gemany Original (German): Ihr Antrag an den Ethikbeirat der Leuphana Universität Lünenurg EB-Antrag Eckert201408_Prokrastination3 Wirkprüfung eines onlinebasierten Trainings gegen Prokrastination Sehr geehrter Dr. Eckert, Ihr oben genannter Antrag wurde am 30.07.2014 eingereicht und vom Ethikbeirat im Umlaufverfahren beraten. Mit dem abschließendem Votum beurteilt der Ethikbeirat die Studie als “ethisch unbedenklich”. Translation (English): Ethics approval Eckert201408_Prokrastination3 Evaluation of an online based training to overcome procrastination Dear Dr. Eckert, your ethics approval was submitted on July 30th 2014 an it was discussed in a circulation procedure by the Ethical Review Board. The final vote of the Ethical Review Board is, there are no ethical concerns with about the study.

## Author Contributions

ME conceived of the presented idea, developed the theory, and performed the computations. DE, DL, and MB verified the analytical methods. DE and BS encouraged ME to investigate the additional effects of SMS-support. All authors discussed the results and contributed to the final manuscript.

## Conflict of Interest Statement

The authors declare that the research was conducted in the absence of any commercial or financial relationships that could be construed as a potential conflict of interest.
